# Acceptability of an Incentivized Peer Referral Intervention to Address COVID-19 Vaccine Hesitancy Among Adults in Yopougon-Est, Côte d’Ivoire

**DOI:** 10.9745/GHSP-D-23-00468

**Published:** 2024-06-27

**Authors:** Katherine Thanel, Brian Pedersen, Yao Kouakou Albert, Mariame Louise Ouattara, Dorgeles Gbeke, Virupax Ranebennur, Holly M. Burke

**Affiliations:** a FHI 360, Seattle, WA, USA.; b FHI 360, Washington, DC, USA.; c University Jean Lorougnon Guédé, Daloa, Côte d’Ivoire.; d Independent research consultant, Abidjan, Côte d’Ivoire.; e FHI 360, Mumbai, India.; f FHI 360, Durham, NC, USA.

## Abstract

**Introduction:**

Vaccine hesitancy persists as a barrier to vaccine uptake among adults across geographies. We pilot-tested an incentivized peer referral intervention in Yopougon-Est, Côte d’Ivoire, to encourage adults who recently received COVID-19 vaccination to discuss their experiences and motivate family and friends to seek vaccination.

**Implementation:** From May through June 2023, the intervention operated at 2 vaccination sites, where staff approached individuals immediately after receiving COVID-19 vaccination. Interested vaccine recipients received up to 9 referral coupons to distribute among their social circles, with a small financial incentive (approximately US$3) offered for each person they referred who returned to 1 of the 2 sites for COVID-19 vaccination.

**Methods:**

We collected data on numbers of people vaccinated and coupons returned. Qualitative interviews were conducted with 40 referred vaccine recipients and 7 public health officials.

**Results:**

During the 6-week intervention, 450 newly vaccinated individuals were offered the opportunity to enroll, with 197 opting to distribute coupons. Nearly half (45%) of these peer mobilizers who distributed coupons referred at least 1 person who subsequently came in for vaccination, and most of this subset had 2 or more completed referrals. Qualitative findings revealed that coupons served as effective reminders, sparking discussions within social networks and prompting vaccine-seeking behavior. According to the referred vaccine recipients, hearing about their peers’ vaccination experience influenced uptake. Vaccine recipients and public health officials found the small referral incentive acceptable. Officials noted the intervention’s potential utility and cost effectiveness, suggesting possible sustainability.

**Conclusion:**

This incentivized peer referral intervention, capitalizing on peer networks and social norms, holds promise for increasing vaccine uptake in Yopougon-Est and potentially in other vaccination contexts globally. Practitioners can leverage the implementation guide and training materials we developed to replicate the intervention at larger scale and assess impact on vaccination trends.

## Abstract (precis)

A pilot intervention in Yopougon-Est, Côte d’Ivoire, found incentivizing recent COVID-19 vaccine recipients to refer peers for vaccination using paper coupons acceptable.

## Teaser

**Key Findings:**

- In response to decreased demand among adults for COVID-19 vaccinations in Yopougon-Est, Côte d’Ivoire, after integrating the vaccines into routine immunization services, an intervention was developed that gave monetary incentives to newly vaccinated individuals when they acted as peer mobilizers and referred family and friends for COVID-19 vaccination with paper coupons.
- Of the 450 vaccinated individuals approached to be peer mobilizers, 197 opted in, distributing up to 1,041 coupons to family and peers. From those coupons, 399 individuals returned for vaccination, for a response rate of 38%.
- Referred vaccine recipients reported the US$3 incentive peer mobilizers received for each referral was socially acceptable, and public health officials reported that this intervention could be integrated into routine vaccination services with relatively low cost and effort.

**Key Implications:**

- Incentivized peer referral interventions may encourage vaccinated individuals to discuss personal experiences to allay rumors and fears that contribute to vaccine hesitancy.
- Practitioners can adapt this incentivized peer referral intervention to other geographies and vaccination settings using the implementation guide, training materials, and data collection tools we developed based on our experience pilot testing this intervention.

## INTRODUCTION

In 2019, the World Health Organization designated vaccine hesitancy a threat to global health.^1^ Hesitancy is the “state of indecision and uncertainty about vaccination before a decision is made”^2^ and does not imply resistance. It persists as a barrier to uptake of COVID-19 and other vaccinations across geographies, including Côte d’Ivoire,^3^ where various public health priorities compete for attention and community support.^4^^,^^5^ While the country reported up to 638 cases per week at one point in the year before the intervention, the data are sporadic,^6^ and evidence suggests that most COVID-19 cases in Côte d’Ivoire were not being detected.^7^ Despite early successes in vaccination, as of September 18, 2022, only 33.43% of Ivorians were fully vaccinated against COVID-19.^8^

The COVID-19: Social Marketing and Behavioral Science Tools and Approaches for Optimizing Throughput at Mass Vaccination Sites in sub-Saharan Africa project was designed to increase demand for COVID-19 vaccination, especially among vaccine-hesitant adults in Yopougon-Est, the country’s largest and most densely populated municipality,^9^ where district officials and their service delivery partner, Village Reach, had recently opened a mass vaccination center. Starting in November 2021, we conducted formative research with students, religious leaders, newly vaccinated individuals, and unvaccinated men and women to identify drivers of COVID-19 vaccine uptake and hesitancy. Insights from this research informed the design of demand-creation activities that were implemented through August 2023. The activities included radio spots and public service announcements, print materials (e.g., posters, flyers) and improved signage for vaccination sites, small trinkets for newly vaccinated individuals (e.g., “I am vaccinated” stickers and bracelets), and mobilization activities implemented by a community-based partner. Community mobilization activities included home visits and small group discussions conducted with established women’s and church groups.

In January 2023, Côte d’Ivoire began to demobilize stand-alone and mobile COVID-19 vaccination services, making the vaccine available in routine vaccination service facilities (call center staff for Centre d’Informations et de Communication Gouvernementale, phone call, September 27, 2023). Site managers in Yopougon-Est reported few requests for COVID-19 vaccination after this integration (site managers at 5 sites considered for implementation, in-person communication, May 2023). District officials requested our project design targeted interventions to address this drop in demand. We began by reviewing our formative research to identify insights that might inform these interventions. A key insight was that while family and peer support for vaccination was one of the most important factors influencing COVID-19 vaccine uptake, few vaccinated individuals routinely discussed their status or experience with others. We concluded that if newly vaccinated individuals could be encouraged to discuss their vaccination status and experience, this may lead to increased uptake among those who were still unvaccinated, especially vaccine-hesitant individuals.

Based on this premise, we adapted a proven peer referral intervention, the Enhanced Peer Outreach Approach (EPOA),^10^ which has been used by the U.S. Agency for International Development’s Meeting Targets and Maintaining Epidemic Control (EpiC) project to increase HIV case identification and linkage to treatment in 21 countries using referral coupons.^11^

The effectiveness of peer referrals to overcome demand-side barriers to uptake of health products and services has been documented in several fields, including family planning,^12^ HIV and sexually transmitted infection control. One study found combining peer referral with monetary incentives for program recipients could promote the distribution of HIV self-testing kits among men who have sex with men in China. This study concluded that peer referral strategies tap into self-identity with a community and trust to provide services more effectively.^13^ Another study reported that incentivizing peer referrals was an effective means of engaging under-represented populations in community-based sexually transmitted infection research and prevention programs, noting the particular relevance in contexts where clinic access is limited.^14^

The effectiveness of peer referrals to overcome demand-side barriers to uptake of health products and services has been documented in several fields.

While some programs have tested incentives for recipients of adult vaccines, such as influenza and tetanus, diphtheria, and pertussis^15^ in addition to COVID-19 vaccination,^16^^,^^17^ interventions incentivizing individual peer referrals for vaccination are less common. Most of these have been designed for research with key populations for Hepatitis B vaccine or HIV and Hepatitis C vaccine trials.^18^^–^^20^ It is difficult to identify interventions that have targeted individuals from the general population as peer mobilizers to promote vaccination within their social networks. A 2022 working paper^21^ suggests that peers play an important role in communicating benefits and dispelling myths about COVID-19 and influenza vaccines, signaling the superior credibility of peers compared with that of qualified medical experts with lower social proximity. In 2022, the New York City Vaccine Referral Bonus Program credited community organizations US$100 for referrals.^22^ However, our intervention is distinct in its approach of directly incentivizing vaccinated individuals to discuss their experiences with and refer peers for vaccination. Identifying novel approaches for addressing vaccine hesitancy among adults is increasingly relevant with improved international availability of newer vaccines, such as human papillomavirus.^23^

## INCENTIVIZED PEER REFERRAL INTERVENTION FOR COVID-19 VACCINATION

We adapted EPOA to incentivize newly vaccinated individuals to refer family members and peers to COVID-19 vaccination services. From May 1, 2023, to June 16, 2023, intervention teams at 2 vaccination sites managed by the Yopougon-Est district health office implemented the incentivized peer referral intervention. They selected facilities in collaboration with local authorities based on estimates of highest volume of COVID-19 vaccinations to increase the chances of quickly identifying a greater number of individuals who could be recruited as peer mobilizers who would distribute referral coupons to their peers. However, at the time, all facilities were experiencing low volume following integration of the COVID-19 vaccine into routine vaccination services and a prolonged stock-out of the Pfizer-BioNTech COVID-19 vaccine, the only vaccine available in the intervention sites during this period.

**FIGURE 1:**
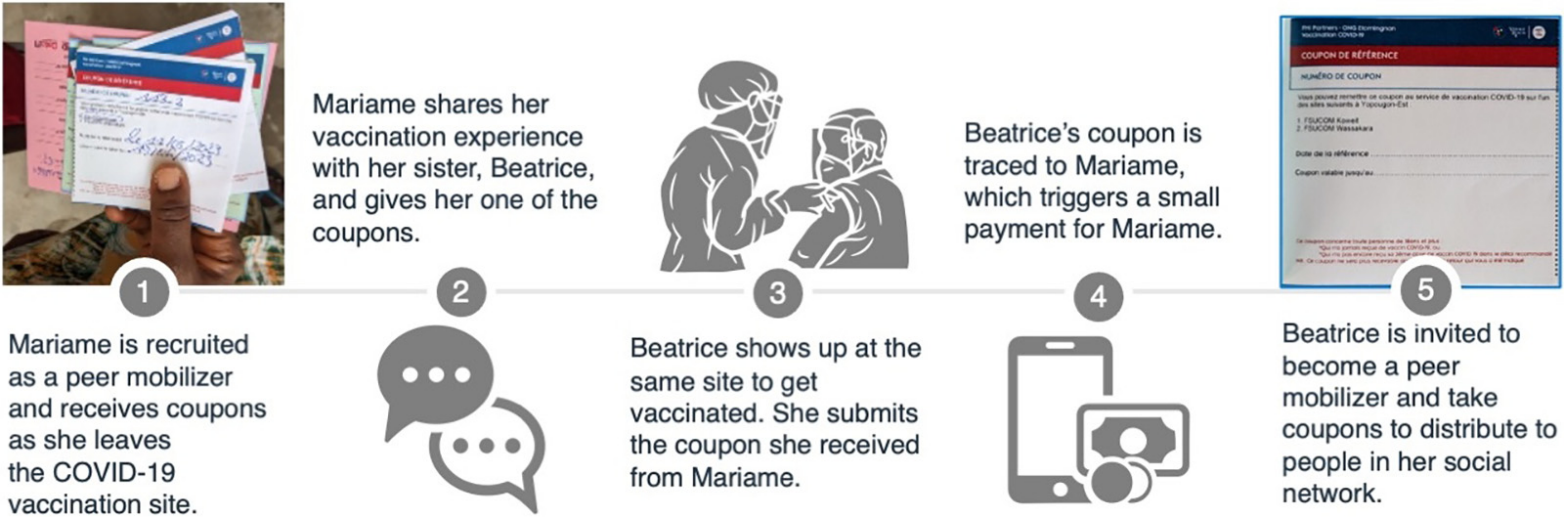
Flow of Incentivized Peer Referral Intervention for COVID-19 Vaccination From a Peer Mobilizer’s Perspective, Mariame

Intervention staff stationed in the facilities approached individuals during their 15-minute post-COVID-19 vaccination observation period and invited them to participate in the intervention as peer mobilizers. Interested individuals were given up to 9 paper referral coupons with information on COVID-19 vaccination sites to distribute in their social network. For each vaccine-eligible adult who returned to 1 of the 2 sites with a coupon during the 8-week intervention, the peer mobilizer received 2000 West African francs (CFA) (approximately US$3) via mobile money. Each paper coupon was marked with a unique number that linked the referred peer to the peer mobilizer to ensure compensation. Individuals who presented at facilities for vaccination spontaneously (without a coupon) and referred peers with a coupon were approached for recruitment to be peer mobilizers. Figure 1 describes the intervention flow from the perspective of a vaccinated individual recruited as a peer mobilizer.

Intervention staff spent a few minutes with each peer mobilizer describing the coupon system, emphasizing the voluntary nature of referrals, and answering questions. They did not train peer mobilizers; the intervention relied on peers sharing their personal experiences as testimony to overcome barriers rather than investing resources in training and requiring them to disseminate key messages. A community-based partner supervised intervention staff.

## METHODS

### Program Data Collection

**FIGURE 2:**
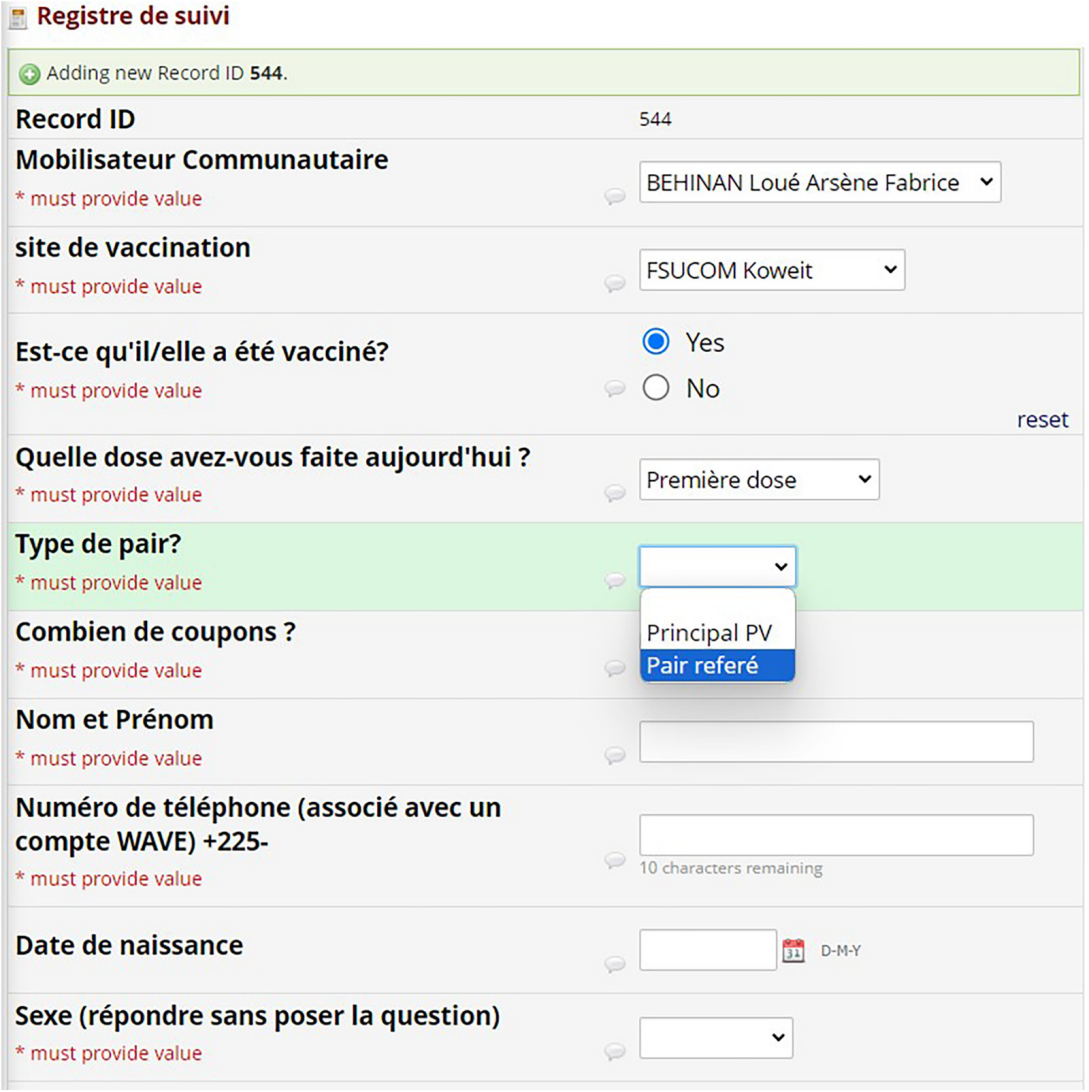
Screenshot of REDCap Registration Data Entry Form

**FIGURE 3:**
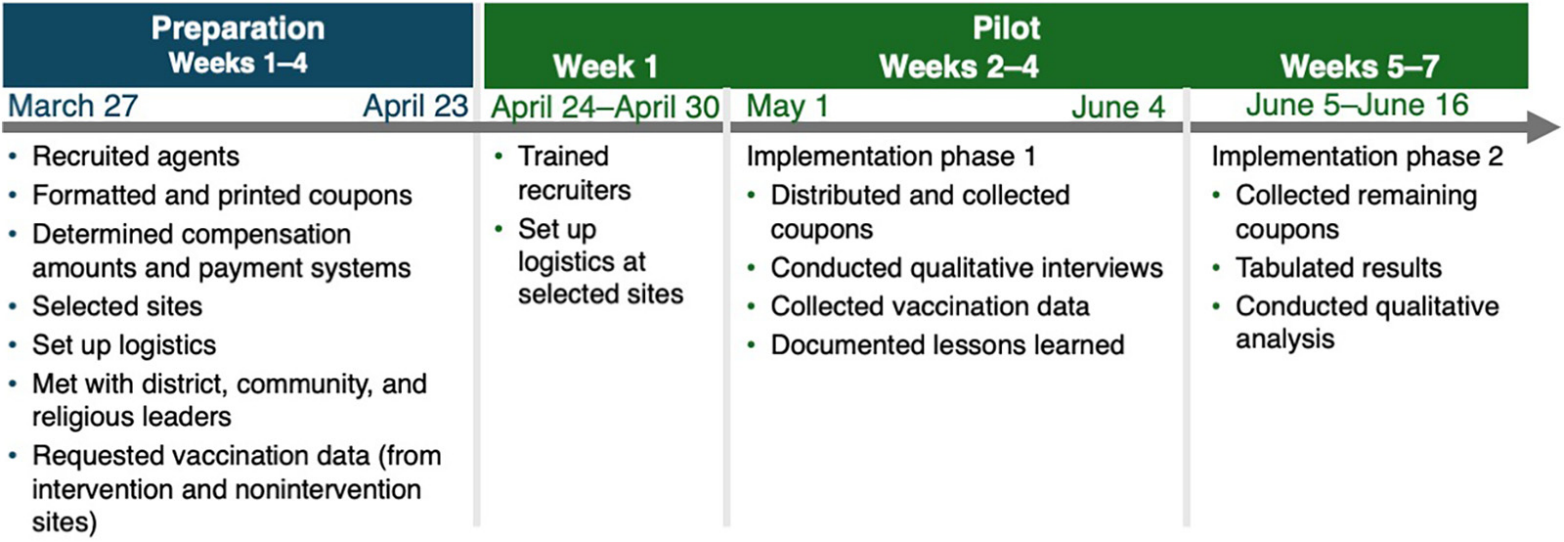
Implementation Timeline of Incentivized Peer Referral Intervention in Yopougon-Est, Côte d’Ivoire

Intervention staff collected data from vaccinated individuals over the 6-week recruitment period for the purposes of registration, recordkeeping, and recording intervention outcomes. Intervention outcomes included number of individuals vaccinated, number who agreed to distribute coupons, primary reason for not wanting to distribute coupons for those who declined, and number of coupons returned (i.e., completed referrals). Staff used a REDCap^24^ data entry form on their mobile phones to collect information on the number of people vaccinated; names, phone numbers, and basic demographic information of people who agreed to be peer mobilizers; whether the peer mobilizer agreed to be contacted in the future about their experience making referrals, and incoming and outgoing coupon codes to assist with coupon tracking and distributing compensation at the end of each week (Figure 2). They assigned coupon codes to individuals who accepted to be peer mobilizers and collected the codes from all referred individuals vaccinated at the 2 sites during the intervention period and for 2 weeks following the intervention, regardless of whether they accepted or declined coupons for distribution (Figure 3). Staff completed a second REDCap form each time they sent a mobile payment. A PowerBI dashboard linked with both REDCap forms enabled the intervention team to monitor networks and track payments made.

**Figure:**
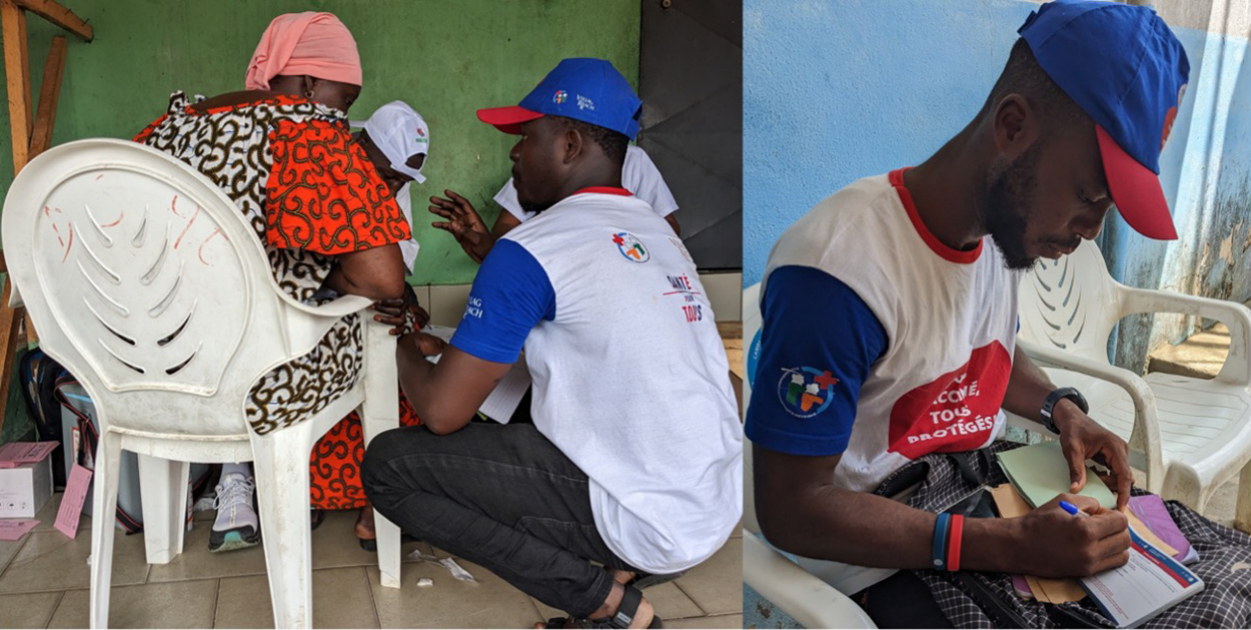
Intervention staff explain the incentivized peer referral program to a newly vaccinated individual and fill out a referral coupon. © 2023 Katherine Thanel/FHI 360; Mariame Lousie Ouattara.

### Qualitative Exploratory Assessment

We designed an exploratory assessment to gather descriptive, preliminary data on the acceptability and potential effects of the incentivized peer referral approach for COVID-19 vaccination to inform potential future rounds of implementation and monitor potential unintended negative consequences of the monetary incentives. The following research questions focused on information that related to the viability of the intervention and that could be used to identify and address problems for program design adaptation.

1. Perceptions of uptake: How does increased awareness of vaccination prevalence and the social proof of learning about peers’ experiences impact uptake?
2. Appropriateness of peer referrals and compensation: How is the approach perceived? Is it appropriate to incentivize referrals in this context? What amount is appropriate? Are people just coming in for the opportunity to earn an incentive?
3. Sustainability of the intervention: Would decision-makers support incentivized peer referral interventions like this one? How can implementation tools be made more user friendly?

We conducted 46 key informant interviews with subsets of different groups, including 29 referred peer mobilizers (individuals who agreed to distribute coupons after being referred themselves for vaccination), 11 referred peers who declined to distribute coupons, and 6 public sector vaccination staff who participated in implementation (2 vaccinators, a registrar, a vaccination service manager, a nurse vaccinator, and a community health volunteer) to explore the first 2 of the 3 sets of research questions. Among the 29 referred peer mobilizers, we interviewed 21 in person immediately following vaccination. The remaining 8 were purposively sampled at the end of the intervention based on their number of completed referrals—4 who did not have any completed referrals and 4 who had 8 or 9 completed referrals—and interviewed by phone.

Finally, we presented programmatic results via a short PowerPoint presentation to 1 district health official to explore our third set of research questions. In this article, we use the term public health officials when referring to perspectives shared by district health official and participating public sector vaccination staff and specify between their perspectives when disparate.

Interviewers worked in pairs: 1 asked the questions using an interview guide and the other took detailed notes to complement audio recordings. The interview team transcribed notes and quotes in Excel data reduction tables organized by interview question and color coded according to the 3 sets of research questions. The research team held weekly data review sessions to discuss data as they were documented and jointly determine when saturation was reached, and interviews could be stopped. We conducted analysis collaboratively through individual reviews followed by group sessions to prioritize the themes that appeared most frequently and those that responded directly to the research questions for reporting. All data were reviewed by 4 of the authors (MO, GD, YA, KT).

### Ethical Approval

This study, IRBNet #2045424, was submitted to FHI 360’s Protection of Human Subjects Committee in April 2023 and was determined to not be research. It was simultaneously approved by the Comité National d’Ethique des Sciences de la Vie et de la Santé in Abidjan.

## RESULTS

### Intervention Outcomes Based on Program Data

**FIGURE 4:**
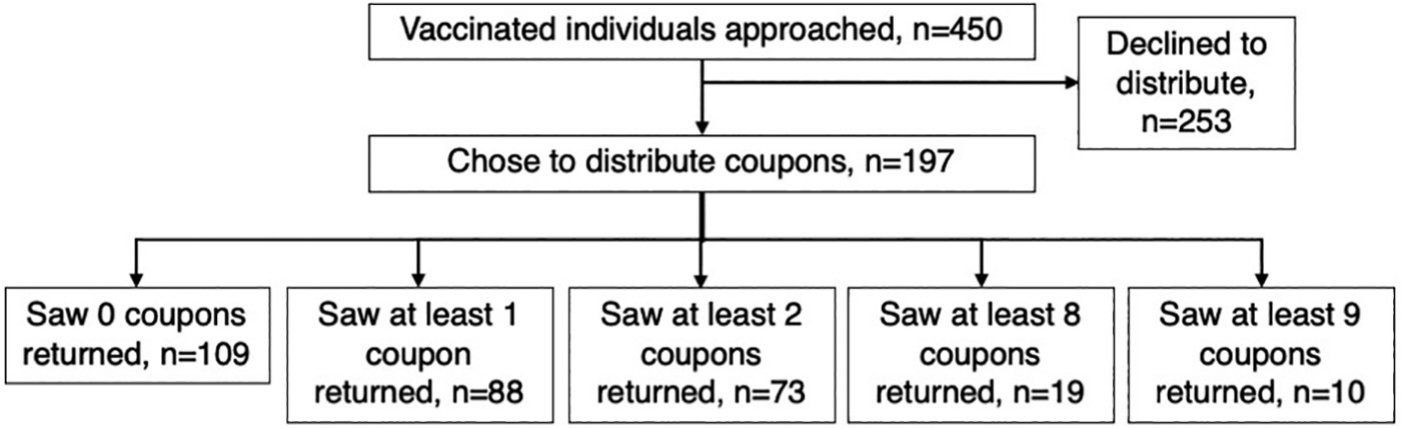
Flow of Intervention Participation and Vaccination Referral Coupons Returned

A total of 450 vaccinated individuals were approached by intervention staff during the recruitment stage (first 6 weeks). Of the 450, 197 individuals (45%) chose to distribute coupons and 253 declined. Of the 1,041 coupons distributed to peer mobilizers, 399 were returned by referred peers, representing a return rate of 38% (Figure 4). This rate is similar to the results of EPOA campaigns implemented by EpiC (Virupax Ranebennur, email, September 25, 2023).

**FIGURE 5:**
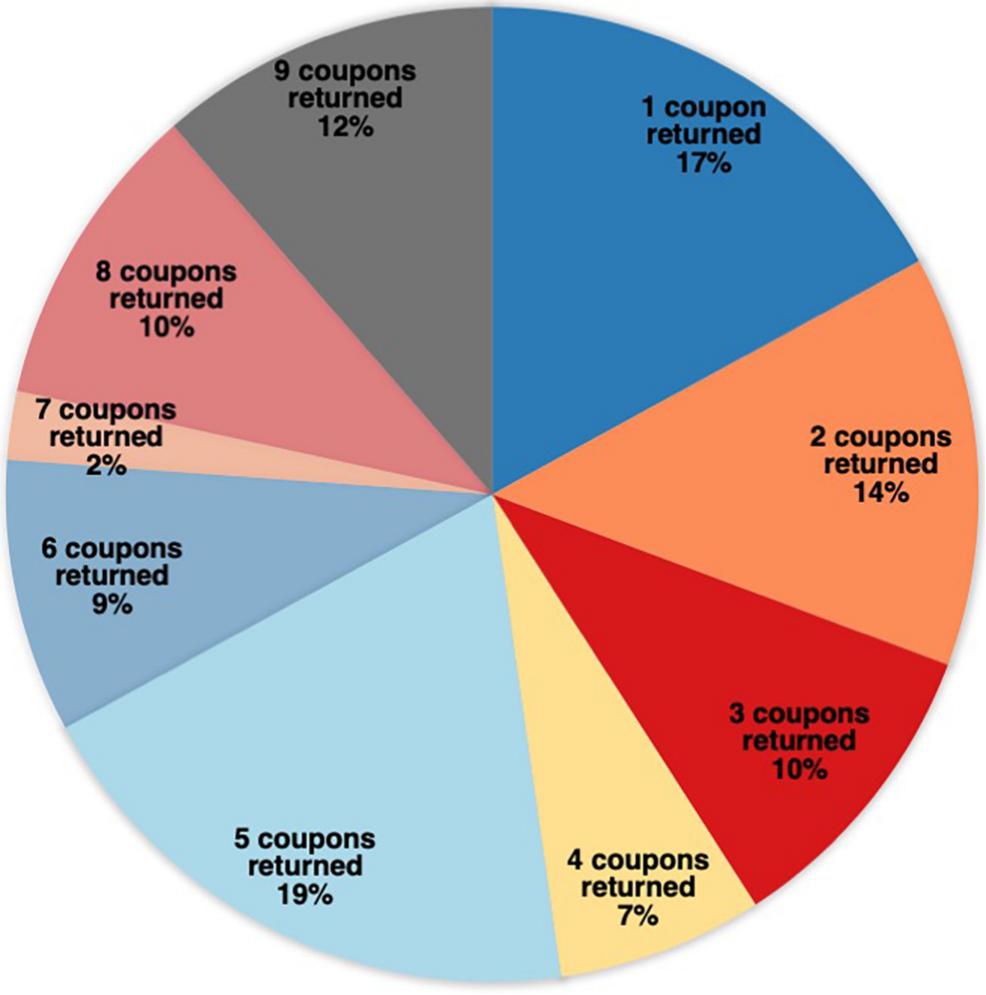
Number of Vaccination Referral Coupons Returned by 88 Successful Peer Mobilizers^a^ ^a^ Mobilizers were limited to a maximum of 9 coupons.

Fewer than half (88, 45%) of the 197 peer mobilizers who chose to distribute coupons saw peers return with coupons for vaccination. Of the 88 that had at least 1 coupon returned, most (83%) had at least 2 coupons returned, over half had 5 coupons returned, and a quarter had 8 or 9 coupons returned (peer mobilizers were limited to 9 coupons) (Figure 5).

We employed PowerBI to display referral networks constructed using coupon codes to link each referred individual to a peer mobilizer. For this intervention, we define referral network as the group of individuals presenting with a coupon referral who are linked to an initial peer mobilizer.

**FIGURE 6:**
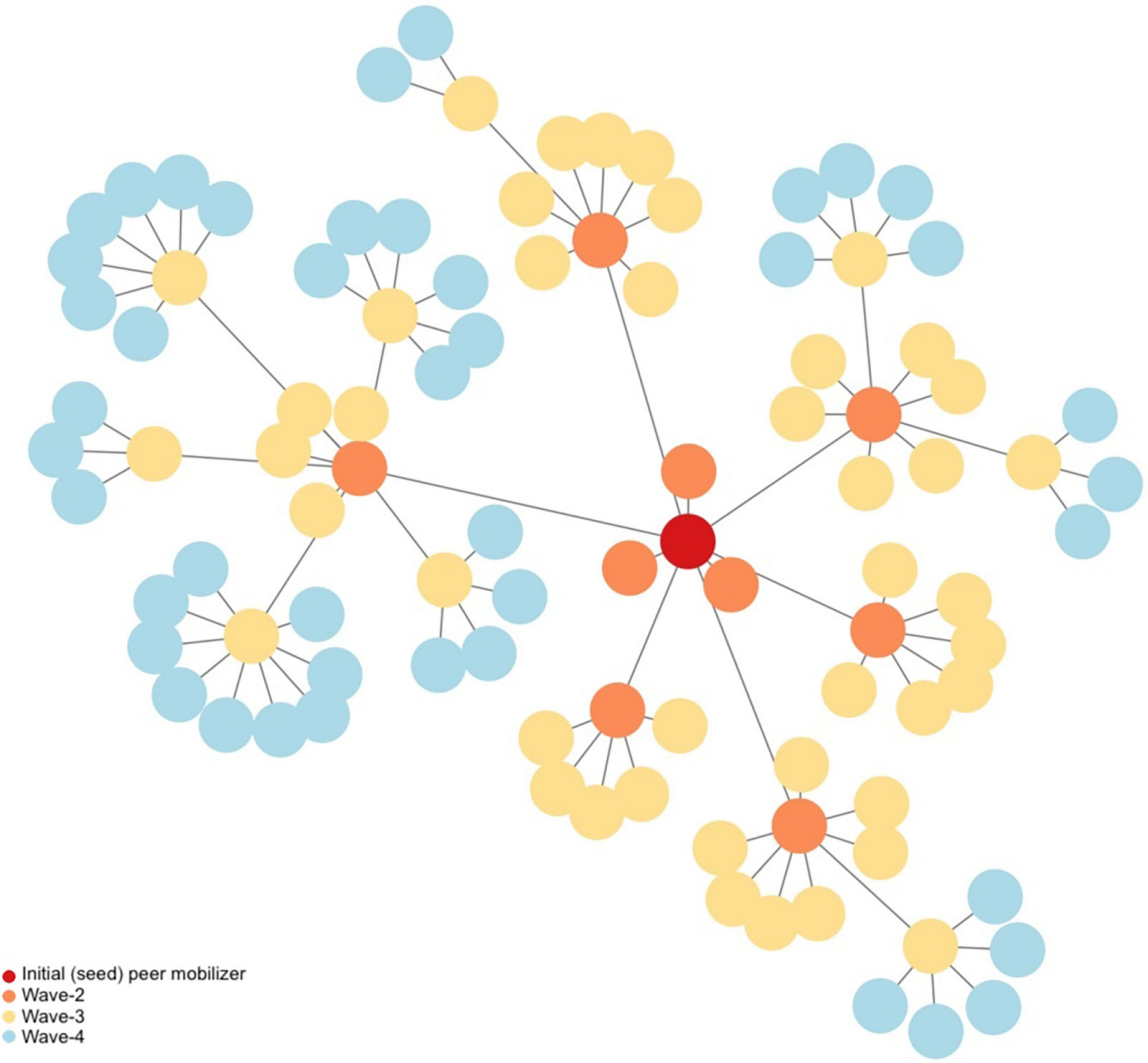
One Referral Network Comprising 90 Referred Peers From One Peer Mobilizer

Of the 399 returned coupons, 70% were associated with networks of 10 or more individuals; 35% were associated with networks of 90 individuals, stemming from only 2 initial peer mobilizers. Figure 6 depicts a referral network of 90 individuals associated with 1 peer mobilizer. Each dot represents an individual vaccinated through the intervention. From the initially recruited peer mobilizer (dark gray dot), each subsequent wave of referred peers is represented with a different color. The lines connecting the dots represent the connections between peer mobilizer and referred peer. This network includes not only peers directly referred by the initial peer mobilizer but also those referred by subsequent waves of peer mobilizers, forming chains of referrals.

Of the 399 returned coupons, 70% were associated with networks of 10 or more individuals, representing those directly referred by the peer mobilizer and those referred by subsequent waves of mobilizers.

**FIGURE 7:**
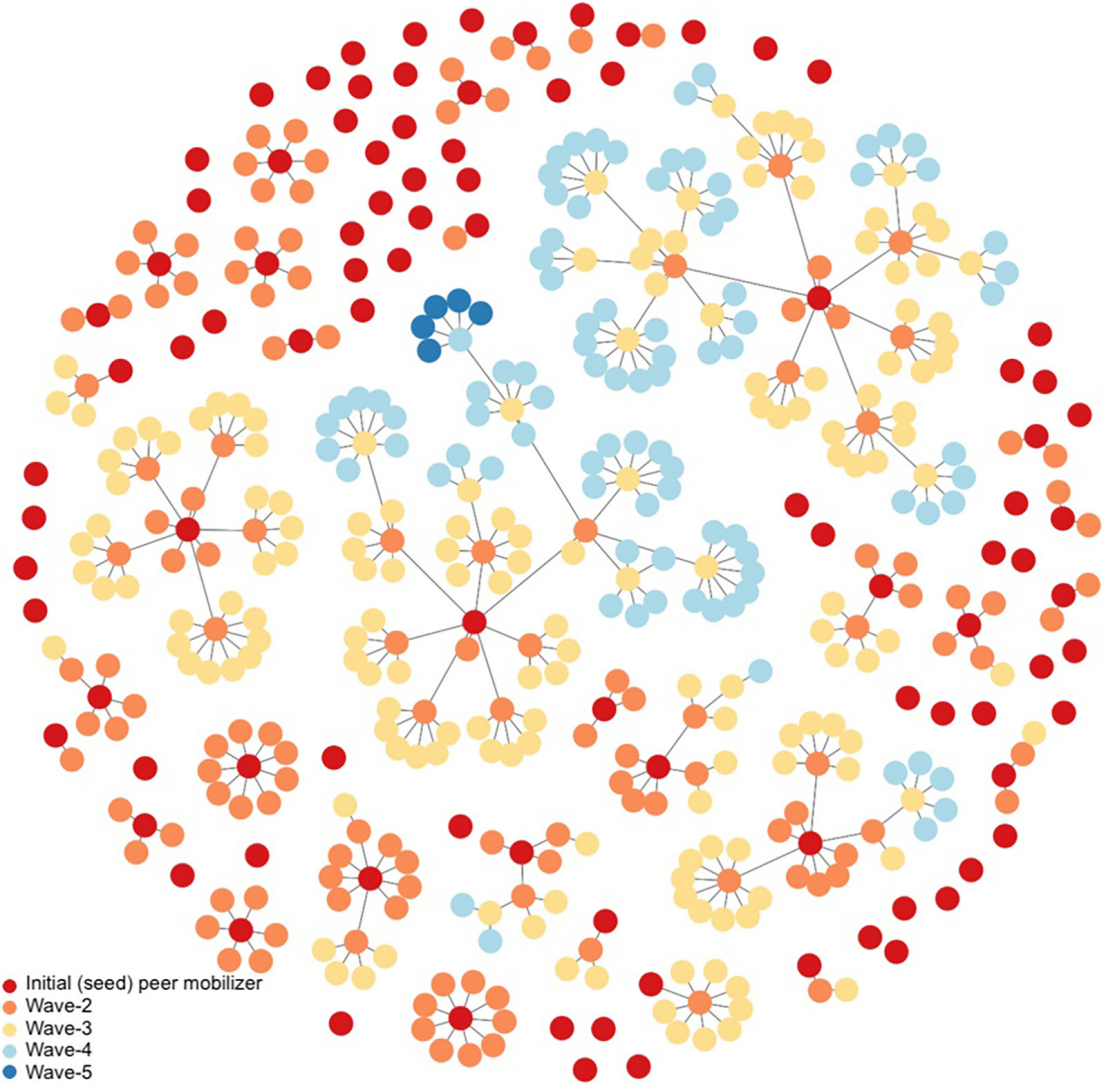
All Intervention Vaccination Referral Networks

More than half (52%) of the returned coupons were associated with the networks of 5 initial peer mobilizers. In Figure 7, each dot represents an individual (n=528) vaccinated over the 8-week intervention period.

**FIGURE 8:**
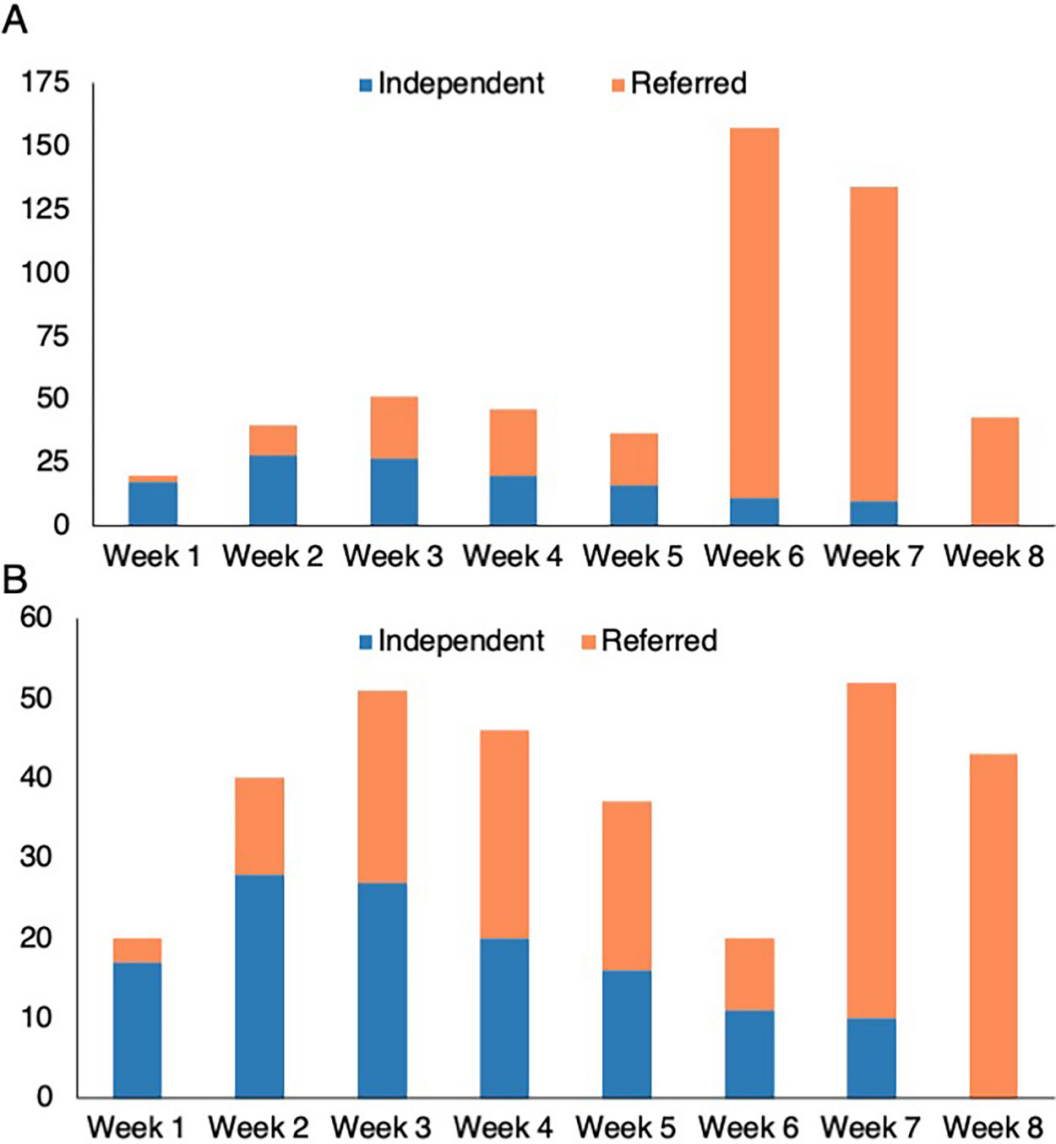
Number of Individuals Vaccinated at Sites During the Intervention Period Who Were Referred and Presented Independently (A) Including Hajj participants; (B) Excluding Hajj Participants^a^ ^a^ We did not collect data on the number of individuals who appeared at the sites independently during week 8 and were not able to obtain data from the sites.

It took 2 weeks for referred peers to begin presenting at a steady rate. In the first few weeks, the sites received more individuals presenting independently for vaccination than those referred by the intervention. From week 4, referred peers exceeded independent appearances. We observed a marked increase of referred peers in weeks 6 and 7 of the 8-week intervention (146 and 124 referrals, respectively) after members of a religious group planning a Hajj (pilgrimage to Mecca) began recruiting peers who came to get vaccinated in preparation for travel (Figure 8). In week 8, after the event, the number of referred individuals returned to about double what we had observed in weeks 3 through 5. When we remove the networks of the Hajj participants, referrals for weeks 6 and 7 are significantly reduced (9 and 42 referrals, respectively). Week 7 referrals are similar to those of week 8 (42 and 43 referrals, respectively). The week of June 16 (intervention week 8), we collected data on referrals but stopped enrolling, thus counting individuals who presented at the sites independently (without a coupon).

We only collected demographic data for individuals who chose to distribute coupons. There was an even split in sex: 99 men and 98 women opted to distribute coupons. Age of peer mobilizers ranged from 18 to 64 years (median=32 years, mean=34 years, interquartile range=23 years, 43 years).

Data on vaccination dose were collected for both referred peers and individuals who arrived at the vaccination center independently without a coupon. Among referred peers, 35% obtained their first dose, 27% their second, 32% their third (booster), and 5.6% their fourth (booster). There was not a marked difference in the percent distribution of doses between the 2 groups.

The most frequent reasons given by referred vaccine recipients not interested in distributing coupons themselves after receiving their vaccine were: “unavailable during implementation period” (52, 15.7%), “all peers have already been vaccinated” (48, 14.5%), “no time to talk today” (38, 11.5%), and “don’t know anyone to recruit” (35, 10.6%). Only 3 (<1%) stated “not comfortable with incentive payment,” and 109 (32.8%) individuals responded with “other.” We did not collect any detail on the “other” reasons due to failure to include an open-ended response option on the data collection form.

Among individuals who chose to distribute coupons, 89% provided their phone numbers and said they would be willing to be contacted to participate in qualitative interviews if selected.

### Key Informant Interviews on Intervention Acceptability and Potential Effects

The vaccinated peer qualitative interview respondents (n=40) ages ranged from 19 to 52 years (median=29 years). We interviewed similar numbers of males and females, 16 and 21, respectively, among those reporting sex. Half of the respondents were Muslim (n=20), 10 were Christians, 1 was Animist, and 9 declined to respond to the question about religion. The skew toward Muslim respondents is likely due more to travel preparations for the Hajj than to neighborhood demographics or other factors. Professions were diverse, including driver, shopkeeper, seamstress, housekeeper, dry cleaner, mechanic, hairdresser, military personnel, cabinet maker, student, and unemployed. Education ranged from no school to university. Half of the participants declined to answer the education question, but among those who responded, most had completed some level of secondary school.

Findings from key informant interviews are organized according to 3 themes aligned with the 3 categories of research questions: perceptions of COVID-19 vaccine uptake, appropriateness, and sustainability of the intervention. The quotes shared under each of these categories are representative of the viewpoints most frequently expressed by respondents. Where opposing views were presented, they are also described.

#### Perceptions of Uptake

The referred vaccine recipients we interviewed reported that the intervention influenced vaccine uptake in 2 primary ways. First, many said the coupons served as prompts, both for the peer mobilizer and referred peer.

*The coupon made my aunt remind me that I haven’t gotten the vaccine yet. She took the opportunity to reassure me about the importance of protecting myself against COVID, which is a real disease. Without the coupon and my aunt’s encouragement, it would have been difficult, if not impossible, for me to come and get vaccinated.* **—**Referred peer

Second, several referred peers reported learning about the experience of vaccinated peers and knowing that people around them were vaccinated reassured and relieved fears related to vaccination.

*My feelings have changed, because before, I was afraid to get vaccinated because they said that when you get vaccinated, you die, they said that the vaccine kills. But my sister told me that this isn’t true. And if she got vaccinated, that means the vaccine is a good thing. So that reassured me to come and do what she did.* **—**Referred peer

Several referred peers reported learning about the experience of vaccinated peers and knowing that people around them were vaccinated reassured and relieved fears related to vaccination.

Many referred vaccine recipients stated that they decided to seek COVID-19 vaccination after hearing about the vaccination experience of the family member or trusted friend who referred them. Several referred peers indicated they trust family members most to provide advice on vaccination.

*One family member can’t suggest something bad to another. Having a family member provide this kind of advice on the COVID-19 vaccine is reassuring.* **—**Referred peer

This perspective on trust was supported by the public sector vaccination staff involved in the intervention.

*It’s true that the community trusts us [the providers]. But that trust isn’t like the trust you have in a family member or a friend. —*Vaccination staff

While we did not collect programmatic data on vaccinated individuals’ relationships to their referring peers, family members were cited more frequently as referrers in the qualitative data than friends, Hajj travel companions, or university classmates, the 3 other groups explicitly mentioned.

#### Appropriateness of Peer Referrals and Compensation

The intervention was well received by all respondents, including the district health official.

*The program is good because it allows a person to think not only of himself but of everyone. If he has understood the importance of the vaccine, he will go and encourage others.*—Peer mobilizer

*Using peers has always been positive here. With all the vaccine gossip, I think using peers has been a good strategy*.—District health official

All participants reported that the modest compensation (approximately US$3) for peer mobilizers was socially acceptable in Yopougon-Est and sufficient to cover local transportation and phone calls related to coupon distribution and follow-up efforts. We did not find evidence implying this amount was coercive.

*2000CFA is enough for transportation, and it’s nice to have thought of paying for transportation for those who live far from the site.* —Peer mobilizer

A couple of participants noted that while they accepted the incentive payment, they would have been willing to participate without compensation. While the district health official found the monetary incentives acceptable for this intervention, he noted that incentives, such as soap, could be a more appropriate alternative in other iterations.

#### Sustainability of Incentivized Peer Referral Interventions

Some vaccination staff said they appreciated that the intervention encouraged the public to seek services rather than obliging providers to conduct continuous outreach to promote vaccination.

*The program has created a link between the center and the community. Community members themselves come to find out about the vaccine. And that’s something we’re not used to seeing in other vaccination programs. —*Vaccination staff

Unlike traditional vaccination campaigns, vaccination staff noted that this intervention reduced mobilization costs as it did not require logistics, such as chairs and tarps, and dedicated outreach staff.

The district health official reported that the intervention may also be more effective than existing vaccine promotion efforts.

*The community isn’t really motivated [to get vaccinated] – our messages aren’t getting through. But with this strategy, I think [the peer mobilizers] can help us do that.—*District health official

## DISCUSSION

This intervention builds on existing evidence demonstrating the role of social networks in influencing individual behavior. Like the experience of EPOA campaigns finding undiagnosed cases of HIV infection, this intervention reached individuals who had not yet been vaccinated against COVID-19 more than 2 years after the vaccine’s introduction in Côte d’Ivoire. The coupon return rate of 38% is considered a success by EPOA campaign standards, and the network results showed a few deep networks representing most of the referrals, resembling those of EPOA campaigns. The incentivized peer referral approach has demonstrated impressive results for HIV testing programs and may also be effective for vaccination programs, especially those targeting adults. We believe this is an important avenue to explore given the absence of systems that bring adults in for vaccination^25^ and the increased availability of lifesaving vaccines, such as those for influenza virus, respiratory syncytial virus, and human papillomavirus^26^ globally.

This intervention reached individuals who had not yet been vaccinated against COVID-19 more than 2 years after the vaccine’s introduction in Côte d’Ivoire.

We designed the intervention in response to formative findings suggesting social support might help overcome vaccine hesitancy. Intervention results suggest this model may be a valuable tool to enhance vaccination coverage, particularly in settings where awareness of peers’ vaccination status is low and community trust and peer influence are significant drivers of vaccine uptake. One such context has been identified in Haiti’s Grand Sud, where practitioners are applying the French version of our implementation guide in an effort to increase uptake of COVID-19 vaccination in a rural setting. Further exploration and larger-scale implementation are warranted to validate our results and assess the potential population-level impact of this intervention on vaccination trends.

### Considerations for Implementation

Our experience provides insights for practitioners interested in replicating this intervention in contexts where hesitancy may affect vaccine uptake. One such insight is that site selection plays an important role in the speed of peer mobilizer recruitment. Facilities in Yopougon-Est were experiencing low throughput for COVID-19 vaccination at intervention outset, and during the first week, only 20 clients received COVID-19 vaccination across the 2 facilities. The low volume of COVID-19 vaccinations meant fewer individuals to recruit and distribute coupons, resulting in a slower start and lower overall numbers than we would have expected at a higher-volume facility. At lower-volume sites, it might be necessary to employ complementary recruitment approaches.

We observed that an incentivized peer referral intervention can produce impressive results even when fewer than half of the individuals who agree to distribute coupons ultimately participate. Our intervention outcomes were the result of the efforts of fewer than 1 in 5 individuals approached and more than half of all referrals stemmed from just 5 prolific peer mobilizers. While program inputs are minimal, with no investment in training peer mobilizers, our experience suggests it may be important to allow the intervention to run for 7 or 8 weeks to identify enough of these highly productive peer mobilizers to observe exponential increases in referrals. We encourage future implementers to plan for a minimum of 8 weeks and examine trends to identify when referrals have peaked.

Our experience suggests it may be important to allow the intervention to run for 7 or 8 weeks to identify enough of these highly productive peer mobilizers to observe exponential increases in referrals.

We employed community mobilizers supervised by a local organization as recruiting agents. While the workload increased over the course of the intervention, at the outset, these mobilizers spent full days at the low-volume sites to engage with only 1 or 2 vaccinated individuals for 10 minutes each. In other low-volume contexts, especially those with flexible payment structures, a service provider could play the role of the recruiting agent.

A consultant hired by FHI 360 was responsible for oversight and verification of returned coupons and payments. This role assured accountability and smooth operation, which would become increasingly important in an opportunity to scale the intervention.

We determined the amount of compensation for peer mobilizers in consultation with implementers and local authorities. The amount was calculated to cover approximate transportation and communication costs associated with coupon distribution and follow-up. Our interviews with referred vaccine recipients, both those who accepted and those who declined to distribute coupons after vaccination, confirmed the appropriateness of the amount. Monetary incentives will not work in every context; appropriateness and potential for abuse were primary themes we explored with our acceptability questions during the pilot. We recommend this process of consultation and monitoring for other programs considering incentivized referrals.

### Limitations

We had planned to conduct a trend analysis using routine vaccination data from before and after the intervention to explore impact on uptake. However, modifications to the national COVID-19 vaccination implementation strategy resulted in changes to the duties and payment structures of the individuals responsible for entering and managing site-level vaccination data. Subsequently, site-level data were not available for the months immediately preceding or following our intervention. Thus, our results rely on program data and qualitative data collected during the intervention. We only collected demographic data for individuals who chose to distribute coupons. In retrospect, we wish we had collected data from all individuals vaccinated during the intervention period to compare the demographics of individuals who were interested with those who declined participation. We recommend this modification in future iterations.

Even without the challenges related to vaccination data access, we recognize that our small sample size, due to human resource constraints, prevented us from attempting to attribute increases in vaccination to our intervention. However, we believe our results are encouraging and the intervention warrants future evaluation in a larger pilot to assess its impact on vaccine uptake.

## CONCLUSION

Implementation of an incentivized peer referral intervention for COVID-19 vaccination in Yopougon-Est, Côte d’Ivoire, produced insights into the potential of referral coupons and small incentives to encourage the sharing of experiences for increased uptake. Overall, this innovative strategy holds promise for improving vaccination coverage and fostering community engagement in vaccination efforts in similar contexts worldwide.

## Notes

**Peer Reviewed**

**First published online:** May 9, 2024.

## References

[1] Ten threats to global health in 2019. World Health Organization. Accessed April 12, 2024. https://www.who.int/news-room/spotlight/ten-threats-to-global-health-in-2019

[2] Larson HJ, Gakidou E, Murray CJL. The vaccine-hesitant moment. N Engl J Med. 2022;387(1):58–65. 10.1056/NEJMra2106441. 35767527 PMC9258752

[3] Ackah BBB, Woo M, Stallwood L, et al. COVID-19 vaccine hesitancy in Africa: a scoping review. Glob Health Res Policy. 2022;7(1):21. 10.1186/s41256-022-00255-1. 35850783 PMC9294808

[4] Cisse A. Analysis of Health Care Utilization in Côte d’Ivoire. African Economic Research Consortium; 2011. Accessed April 12, 2024. https://publication.aercafricalibrary.org/server/api/core/bitstreams/33c7494f-75e8-4656-815d-2a28c992967d/content

[5] Pedersen B, Thanel K, Kouakou A, et al. Identifying drivers of COVID-19 vaccine uptake among residents of Yopougon Est, Abidjan, Côte d’Ivoire. Vaccines (Basel). 2022;10(12):2101. 10.3390/vaccines10122101. 36560511 PMC9783544

[6] Number of COVID-19 cases reported to WHO, Côte d’Ivoire. World Health Organization COVID-19 Dashboard. Accessed April 12, 2024. https://data.who.int/dashboards/covid19/cases?m49=384

[7] Burki TK. Undetected COVID-19 cases in Africa. Lancet Respir Med. 2021;9(12):e121. 10.1016/S2213-2600(21)00504-X. 34774187 PMC8585486

[8] Daily COVID-19 vaccine doses administered. Côte d’Ivoire. Our World in Data. Accessed April 12, 2024. https://ourworldindata.org/covid-vaccinations

[9] Institut National de la Statistique. Recensement Général de la Population et de L’habitation. Institut National de la Statistique; 2021.

[10] Lillie TA, Persaud NE, DiCarlo MC, et al. Reaching the unreached: performance of an enhanced peer outreach approach to identify new HIV cases among female sex workers and men who have sex with men in HIV programs in West and Central Africa. PLoS One. 2019;14(4):e0213743. 10.1371/journal.pone.0213743. 30943205 PMC6447144

[11] Lillie T. Implementing an enhanced peer outreach approach to expand reach of HIV services among key populations. FHI 360/R&E Search for Evidence. August 7, 2019. Accessed April 12, 2024. https://researchforevidence.fhi360.org/implementing-an-enhanced-peer-outreach-approach-to-expand-reach-of-hiv-services-among-key-populations

[12] Flanagan S, Gorstein A, Nicholson M, et al. Behavioural intervention for adolescent uptake of family planning: a randomized controlled trial, Uganda. Bull World Health Organ. 2021;99(11):795–804. 10.2471/BLT.20.285339. 34737472 PMC8542266

[13] Zhou Y, Lu Y, Ni Y, et al. Monetary incentives and peer referral in promoting secondary distribution of HIV self-testing among men who have sex with men in China: a randomized controlled trial. PLoS Med. 2022;19(2):e1003928. 10.1371/journal.pmed.1003928. 35157727 PMC8887971

[14] Campbell MB, Ratnayake A, Gomes G, Stoecker C, Kissinger PJ. Effectiveness of incentivized peer referral to increase enrollment in a community-based chlamydia screening and treatment study among young black men. J Racial Ethn Health Disparities. 2023;1-9. 10.1007/s40615-023-01595-5. 37095285 PMC10124922

[15] Dudley MZ, Taitel MS, Smith-Ray R, Singh T, Limaye RJ, Salmon DA. Effect of educational and financial incentive-based interventions on immunization attitudes, beliefs, intentions and receipt among close contacts of pregnant women. Vaccine. 2021;39(6):961–967. 10.1016/j.vaccine.2020.12.067. 33423837

[16] Bennett NG, Bloom DE, Ferranna M. Factors underlying COVID-19 vaccine and booster hesitancy and refusal, and incentivizing vaccine adoption. PLoS One. 2022;17(9):e0274529. 10.1371/journal.pone.0274529. 36136997 PMC9498968

[17] Rosen AD, Beltran J, Thomas E, et al. COVID-19 vaccine acceptability and financial incentives among unhoused people in Los Angeles County: a three-stage field survey. J Urban Health. 2022;99(3):594–602. 10.1007/s11524-022-00659-x. 35639229 PMC9153868

[18] Magalhães RDLB, Carvalho VM, Brito GMI, Oliveira LB, Galvão MTG, Gir E. Risk practices and immunization against hepatitis B among female sex workers. Revista da Rede de Enfermagem do Nordeste. 2016;17(5):636. 10.15253/2175-6783.2016000500008

[19] Coombs A, McFarland W, Ick T, Fuqua V, Buchbinder SP, Fuchs JD. Long-chain peer referral to recruit black MSM and black transgender women for an HIV vaccine efficacy trial. J Acquir Immune Defic Syndr. 2014;66(4):e94–e97. 10.1097/QAI.0000000000000197. 24815850 PMC4118575

[20] White B, Madden A, Prins M, et al. Assessing the feasibility of hepatitis C virus vaccine trials: Results from the Hepatitis C Incidence and Transmission Study-community (HITS-c) vaccine preparedness study. Vaccine. 2014;32(42):5460–5467. 10.1016/j.vaccine.2014.07.091. 25131726 PMC4509604

[21] Alsan M, Eichmeyer S. Experimental evidence on the effectiveness of nonexperts for improving vaccine demand. Am Econ J Econ Policy. 2024;16(1):394–414. 10.1257/pol.20210393. 38433953 PMC10907065

[22] Cohn E, Chimowitz M, Long T, Varma JK, Chokshi DA. The effect of a proof-of-vaccination requirement, incentive payments, and employer-based mandates on COVID-19 vaccination rates in New York City: a synthetic-control analysis. Lancet Public Health. 2022;7(9):e754–e762. 10.1016/S2468-2667(22)00196-7. 36057274 PMC9433052

[23] Closing the gap: UNICEF bolsters country efforts to increase HPV vaccination. UNICEF. April 28, 2023. Accessed April 12, 2024. https://www.unicef.org/supply/stories/closing-gap-unicef-bolsters-country-efforts-increase-hpv-vaccination

[24] REDCap Data Collection Tool and Database. Accessed April 12, 2024. https://www.project-redcap.org/

[25] World Health Organization (WHO). Immunization Agenda 2030: A Global Strategy to Leave No One Behind. WHO; 2020. Accessed April 12, 2024. https://www.who.int/docs/default-source/immunization/strategy/ia2030/ia2030-document-en.pdf 10.1016/j.vaccine.2022.11.042 39004466

[26] WHO updates recommendations on HPV vaccination schedule. News release. World Health Organization; December 20, 2022. Accessed April 12, 2024. https://www.who.int/news/item/20-12-2022-WHO-updates-recommendations-on-HPV-vaccination-schedule#:∼:text=WHO%20now%20recommends%3A

